# Enhancing understanding and improving prediction of severe weather through spatiotemporal relational learning

**DOI:** 10.1007/s10994-013-5343-x

**Published:** 2013-04-13

**Authors:** Amy McGovern, David J. Gagne, John K. Williams, Rodger A. Brown, Jeffrey B. Basara

**Affiliations:** 1 School of Computer Science, University of Oklahoma, Norman, OK 73019 USA; 2 School of Meteorology, University of Oklahoma, Norman, OK 73072 USA; 3 Research Applications Laboratory, National Center for Atmospheric Research, Boulder, CO 80301 USA; 4 NOAA/National Severe Storms Laboratory, Norman, OK 73072 USA

**Keywords:** Statistical relational learning, Spatiotemporal, Severe weather

## Abstract

Severe weather, including tornadoes, thunderstorms, wind, and hail annually cause significant loss of life and property. We are developing spatiotemporal machine learning techniques that will enable meteorologists to improve the prediction of these events by improving their understanding of the fundamental causes of the phenomena and by building skillful empirical predictive models. In this paper, we present significant enhancements of our Spatiotemporal Relational Probability Trees that enable autonomous discovery of spatiotemporal relationships as well as learning with arbitrary shapes. We focus our evaluation on two real-world case studies using our technique: predicting tornadoes in Oklahoma and predicting aircraft turbulence in the United States. We also discuss how to evaluate success for a machine learning algorithm in the severe weather domain, which will enable new methods such as ours to transfer from research to operations, provide a set of lessons learned for embedded machine learning applications, and discuss how to field our technique.

## Motivation and introduction

The long-term goal of our research is to fundamentally transform scientists’ understanding and prediction of severe weather phenomena through the development and application of spatiotemporal machine learning/data mining techniques. Severe weather phenomena, including tornadoes, thunderstorms, hail, and wind, annually cause significant loss of life and property (e.g., $32B in the United States in 2011, Lubber 2012). Thunderstorms produce turbulence that is dangerous to aviation, causing costly diversions, delays, cancellations, and occasional accidents (Eichenbaum 2003). Improving the prediction of such events will have an immediate impact to society.

**Fig. 1:**
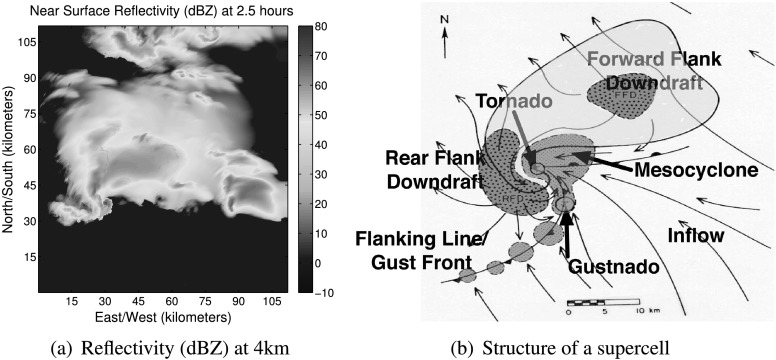
These figures are best viewed in color. (**a**): Simulated reflectivity (proportional to precipitation intensity) just above the ground. (**b**): Structure of a classic supercell (adapted from Lemon and Doswell 1979; Davies-Jones 1986; Bluestein 1993)

Humans are very good at pattern recognition, including scientific discovery. However, humans have difficulty processing the overwhelming amount of data being produced by weather observations and numerical models. Meteorologists rely on conceptual models to help them when they issue severe weather warnings (Lemon and Doswell 1979; Rasmussen 2003). Although severe weather events are continuous, dynamic entities, meteorologists study them through discrete high-level features and relationships. For example, Fig. 1(a) shows the simulated reflectivity 25 m above the ground. Figure 1(b) shows the structure of a canonical supercell thunderstorm (e.g., Lemon and Doswell 1979; Davies-Jones 1986; Bluestein 1993). Comparing the lower left portion of Fig. 1(a) to Fig. 1(b), we can see a hook echo (a comma shaped region of high reflectivity) that, coupled with the low reflectivity region of inflowing air adjacent to it, indicates a region of rotation (a mesocyclone). The inflow converging into the low reflectivity region produces a strong rotating updraft (air flowing upward). A hook echo is an indicator of a potential tornado.

We are using statistical relational learning (SRL, Jensen and Getoor 2003; Fern et al. 2006; Getoor and Taskar 2007) to study these phenomena, which enables machine learning to build models of the data using objects (e.g., the high level features identified by the meteorologists) and the relationships between them. These relationships are crucial. For example, the relationship between the updraft and the rear-flank downdraft, an area of relatively cooler and drier air that spreads out behind the storm, is thought to play a significant role in the creation of tornadoes (e.g., Rotunno 1993). SRL has proven to be very successful in a wide variety of applications (e.g., Neville et al. 2005; Fast et al. 2007; Neville and Jensen 2007; Raghavan et al. 2012). We previously developed the Spatiotemporal Relational Probability Tree (SRPT) and its related Spatiotemporal Relational Random Forest (SRRF) techniques (McGovern et al. 2008, 2010, 2011, 2013) and have demonstrated that they can be successfully applied to severe weather applications.

For this special issue focusing on machine learning with importance to society and science, we summarize our work in developing spatiotemporal machine learning methods and applying them to severe weather data. We introduce several significant enhancements to the SRPT and SRRF. We focus on an analysis of two case studies of different severe weather phenomena, a discussion of how to verify machine learning methods on severe weather, an impact discussion from several meteorologists, a discussion of how to field these techniques, and lessons learned for embedding machine learning in a real-world application.

## Related work in meteorology

The environment within which tornadic storms form is well recognized and is used to issue tornado watches by the National Weather Service’s (NWS’s) Storm Predication Center (e.g., Johns and Doswell 1992; Moller et al. 1994; Thompson et al. 2007). However, once storms form, it is difficult to identify which storms will produce tornadoes. The most severe tornadoes develop within supercell thunderstorms that are detectable using the NWS network of Doppler weather radars (e.g., Brown et al. 1978). These radars measure reflectivity and Doppler velocity (component of precipitation particle motion relative to the radar viewing direction) as well as newly-added dual-polarization data (used to deduce precipitation particle type and size) within the storms. Short-term tornado warnings typically are based on the presence of a supercell thunderstorm using radar information. Unfortunately, most tornado warnings are false alarms (e.g., Simmons and Sutter 2011) because only a minority of supercell storms produce tornadoes and there are no unique radar or visual signatures that distinguish these storms.

Meteorologists use numerical modeling of supercell storms with the goal of discovering precursors that will help discriminate between tornadic and nontornadic supercell storms (e.g., Klemp and Rotunno 1983; Wicker and Wilhelmson 1995; Snook and Xue 2008). Numerically-modeled storms typically are initiated by letting a bubble of warm air rise and interact with vertical profiles of wind, temperature, and moisture that are similar to those typically found in tornadic supercell environments. Though idealized, output from a numerical model provides an evolving three-dimensional picture of the temperature, humidity, wind, and hydrometeor particles (rain, hail, graupel, snow) within the modeled storm. One of the important parameters that is computed from the three-dimensional wind field is the vertical component of vorticity^1^ (rotation about a vertical axis), because it is the concentration of vorticity within the storm’s mesocyclone that leads to a tornado-scale vortex (e.g., Markowski et al. 2003; Davies-Jones 2008).

Atmospheric turbulence is a significant source of concern for airline dispatchers, air traffic managers and pilots. Avoiding turbulence is a priority for ensuring passenger safety and comfort, yet unwarranted cancellations, delays and deviations can be costly in time, staff compensation and fuel use, not to mention the disrupted plans of passengers. The Federal Aviation Administration (FAA) has begun addressing this issue by sponsoring development of a gridded turbulence forecast product known as Graphical Turbulence Guidance (GTG, described in Sharman et al. 2006). Operational numerical weather prediction (NWP) models such as the Weather Research and Forecasting (WRF) Rapid Refresh (Benjamin et al. 2006; Skamarock and Klemp 2008) do not yet create forecasts at a scale that can explicitly resolve wind motions that comprise turbulence affecting aircraft (10s to 100s of meters), so GTG relies on a combination of “diagnostics” that infer turbulence from gradients and statistics from the 3-D forecast fields. This approach works reasonably well for clear-air turbulence and mountain-wave turbulence. However, it significantly lacks in its ability to diagnose turbulence in and around thunderstorms, where turbulence can be particularly dynamic and intense. This so-called convectively-induced turbulence (CIT) may be produced by the shears associated with updrafts, downdrafts, storm tops penetrating the tropopause,^2^ or gravity waves^3^ that travel away from the storm and may “break” like waves on a beach. It is a result of complex interactions between the storm dynamics and environment (Lane et al. 2012). In an attempt to mitigate the CIT hazard, FAA guidelines (FAA 2012) currently call for pilots to avoid thunderstorms by a wide margin. However, flight track data show that these guidelines are frequently violated, either because the pilot is unaware of the proximity of the storm or because other considerations (e.g., low fuel or a destination near the storm) make following them untenable. A better understanding of the relationship between radar, satellite and lightning observations, NWP model forecasts, and CIT is required in order to better utilize available information to give pilots automated, specific, actionable guidance on which airspace is likely to be hazardous.

## Spatiotemporal relational probability trees/forests

We have previously introduced Spatiotemporal Relational Probability Trees (SRPT) and their associated ensemble forests (SRRF) (McGovern et al. 2008, 2010, 2011, 2013). In this paper, we focus on the new aspects. The full details of how to grow the trees and the forests are described in Appendix A. We omit the low-level details in the main body of the paper and focus on the high-level discussion of what is new along with a brief overview, necessary to understand these new features.

### Spatiotemporal relational attributed data

Traditional decision trees such as C4.5 (Quinlan 1993) use propositional data, which consist of a series of attribute-value pairs. Although we could represent severe weather data in this manner, we would not be able to reason about or autonomously discover relationships between the high-level features using such a representation. Instead, we use an enhanced version of the relational attributed graph representation developed by Neville et al. (2003).

Relational data contain *objects*, such as high-level concepts that meteorologists already use to describe the data and *relationships* between these objects. In the previous work, we enabled objects to have spatiotemporally varying *fields* of scalar and vector data associated with them. We call these *fielded objects*, following the convention in geographic information systems (Goodchild et al. 2007; Cova and Goodchild 2002). We have previously described our modifications in McGovern et al. (2010, 2011, 2013) and we briefly describe the data through an example here.

**Fig. 2:**
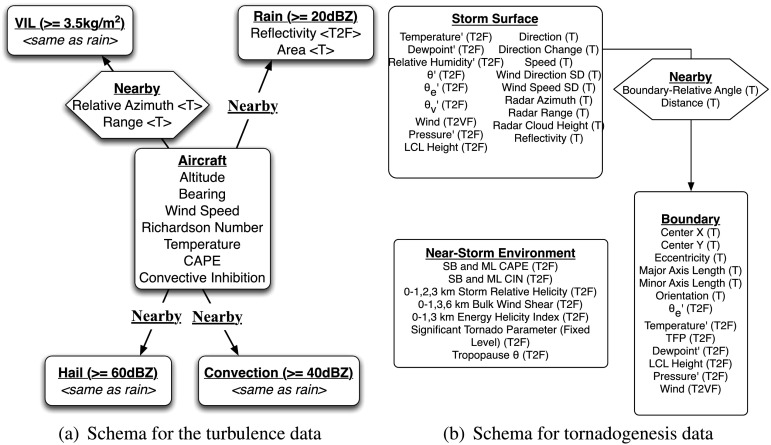
Schema for the (**a**) aircraft turbulence and (**b**) tornadogenesis data

Figure 2 shows the *schema* for data that we have used to predict (a) aircraft turbulence associated with nearby storms and (b) the formation of tornadoes. These data are fully described below. We use them here to illustrate spatiotemporal relational attributed data. Each object, such as an aircraft or a region of precipitation such as rain or hail, is shown in the schema with a rounded box. For example, there are five types of objects that can appear in the turbulence graphs. Although each graph can only have one aircraft object, the other four types may appear more than once, depending on the storms surrounding the aircraft. The pre-specified relationships are shown with hexagons and describe possible spatial relationships between the aircraft and the precipitation regions. Objects and relationships can each have attributes associated with them. These attributes can be *static*, meaning they don’t change during the lifetime of the object, or *dynamic*. Univariate temporally varying attributes are denoted with a T and two or three dimensional fields are denoted as T2F or T3F respectively.

In the work described here, we also enable the data to be described through objects only and do not require the domain scientist to pre-specify the list of possible relationships. This is important as the domain scientist may not be able to mathematically specify some of the complicated relationships, such as the idea of a downdraft wrapping around an updraft. In future work, we would also like to enable object discovery. Currently, we include all objects that could be important based on years of study of the atmosphere.

### Spatiotemporal relational probability trees and forests

Spatiotemporal Relational Probability Trees (SRPTs) are probability estimation trees (Provost and Domingos 2003) that learn with spatiotemporally varying relational data. We give a brief overview here and the full details of the learning algorithm are provided in Appendix A. SRPTs differ from existing tree-based relational learning approaches such as TILDE (Blockeel and De Raedt 1998; Ramon et al. 2002) in their ability to handle the discovery of multi-dimensional relationships (such as the spatial ones introduced here) and their ability to handle temporally varying data. SRRF also differs from the Relational Probability Tree (RPT Neville et al. 2003) and the temporal extensions to the RPT (Sharan and Neville 2007, 2008) in its ability to handle spatially and spatiotemporally varying relational data. This is critical for applications to severe weather.

A single SRPT is grown using the standard greedy algorithm from decision trees such as ID3 and C4.5 (Quinlan 1986, 1993). Since the trees are primarily used in a forest grown using the same randomization and bagging approach as Random Forests (Breiman 2001), the learning algorithm does not prune. Instead of asking questions about each attribute/value pair at a tree node, the SRPT can ask spatiotemporal questions based on a series of templates that we have developed. The full list of possible questions is given in Appendix A. We give illustrations of the questions below.

Data are split in the tree through questions. A question similar to those used by C4.5 trees could be “Is there an updraft with a maximum vertical wind speed of at least 30 m s^−1^?” The specific thresholds that appear in each question are chosen using sampling on the training data. In previous work, we examined the sensitivity of the performance to these numbers. The domain scientists prefer us to report a range of numbers rather than a specific one. In current work, we are investigating the best way to identify this range across the forest and to communicate it to the domain scientists.

An example of a temporal question is “Is the partial derivative (computed using finite differences) of the area of the storm object ≥2 within 5 minutes?” This type of question enables the data to be split on the growth or shrinkage of objects during the storm. Other temporal questions enable the data to be split based on sustaining a value for a certain amount time or on statistics of how the values change over time.

Because we focus on severe weather data, we have enhanced the SRPT to include questions about wind fields that are important to the formation of severe weather. These questions examine how the wind field is converging or diverging in the neighborhood of the storm as well as measuring the instantaneous spin.

The objects in the severe weather data are either two or three dimensional, depending on the source of the data. In both cases, they take on a variety of shapes but they rarely take on a canonical shape such as a circle, cylinder, or cone. In previous work, we had implemented a shape recognition algorithm for such shapes (McGovern et al. 2013) but it limited the identifiable shapes.

We have now developed two approaches that can distinguish arbitrary shapes. For two dimensional data, we use shapelets as developed by Ye and Keogh (2009), Mueen et al. (2011). Shapelets are pieces of a time-series that can be used to distinguish different time series. We use the method from Keogh et al. (2006) to convert two dimensional shapes to time series. The template for this type of tree node question is “Does the temporal shapelet of array attribute *a* on item of type *t* match in this graph?” The shapelet used for comparison is chosen from the training data. The new shapelets are one of the most frequently chosen questions by the tree. In the tornadogenesis data described below, 11 % of the questions in our forests use shapelets.

Three-dimensional shapes cannot be easily reduced to a single time-series and so we use another method to address these types of data. Shape distributions are statistical distributions that characterize a 3D shape (Osada et al. 2002). These can be formed by sampling from random points on the surface of the shape and calculating a simple statistic, such as the distance between the two points. We use this idea to distinguish shapes from one another, by asking the following question: “Given a shape distribution template, is this shape’s distribution statistically the same?” The distributions are distinguished using Kolmogorov-Smirnov. We also distinguish graphs based on a shape changing over time.

In addition to the ability to distinguish arbitrary shapes, the other major enhancement to the SRPT is to enable it to autonomously discover 3D spatial relationships in the data. Spatial relationships are represented using an idea similar to shape distributions. Instead of sampling from two points on the same object, the distribution is created by sampling from one point on each object. This characterizes the shape of the space between the two objects, enabling us to identify such relationships as one object “partially wrapped around” another object, which occur in tornadic storms.

## Verification: moving from research to operations

For a prediction algorithm to be useful in an operational environment, it needs to provide skilled predictions that are physically realistic and consistent. To evaluate these criteria, we use both objective verification scores and subjective evaluation of case studies. Verification scores provide a means to compare the aggregate forecasts with baseline forecasts and to establish the degree of improvement provided by the new system. Case studies allow for an in-depth physical examination of the forecasts so that researchers can discover spatial and temporal tendencies in the forecast and analyze how closely they match the tendencies of the predicted phenomenon.

The verification scores used to evaluate the SRRF focus on its ability to discriminate between two outcomes. The Area Under the Receiver Operating Characteristic (ROC) Curve (AUC; Mason 1982) evaluates how well the algorithm distinguishes between two classes over a range of thresholds throughout the distribution of the forecast values. AUC ranges from 0 to 1 with any value above 0.5 indicating a skilled prediction compared to a random prediction. Binary contingency tables are created at each threshold and can be used to derive a range of scores (Wilks 2011). For this work, we also use the Peirce Skill Score (PSS; Peirce 1884; Hansen and Kuipers 1965) because it can be used to guide the choice of threshold on the ROC curve. All of the verification statistics are defined precisely in Appendix B. The probability threshold with the highest PSS balances the proportion of misses and false alarms, but the ultimate choice of threshold is up to the domain scientist. This is a critical reason for an interdisciplinary approach because the decision threshold chosen by the computer scientist may not be the best choice for a domain where false alarms have a very high cost. We use these scores to evaluate the overall performance of the new learning techniques and to determine how performance varies under different conditions.

The skill statistics appropriate to a particular verification task may depend on the culture of the problem domain and idiosyncrasies of the data available for performing the verification. For instance, in the turbulence domain, ROC AUCs have been used for the FAA’s evaluation of turbulence forecast algorithms before they are made operational (Wandishin et al. 2011). This is appropriate because the AUC is not dependent on the ratio of “true” and “false” events, which is a function of how well pilots avoid turbulence encounters. A different skill statistic such as Critical Success Index (Schaefer 1990) might easily show declines over time as pilots use the turbulence forecasts to avoid turbulence, making it difficult for new turbulence forecasts to show benefit.

Case studies represent an important means for domain experts to evaluate the abilities of new techniques in the context of particular events. Individual case study events can be selected for their ability to test how the new technique handles the evolution of a particular phenomenon (Schultz 2010). Analysis of the output from the technique compared with observations shows how well the technique captured the physical ingredients for a particular situation. Developing the case study output also aids in the process of transitioning the new techniques from research to operations. For each of our domain areas, we are compiling representative case studies for the physical evaluation process. We discuss a few of them in the following section.

Severe weather presents another challenge for machine learning: unbalanced data. Although events such as tornadoes or turbulence are destructive and may seem frequent in the age of constant news coverage, they are quite rare. Pilots do their best to avoid turbulence, which reduces our verified cases of turbulent events. For example, in the aircraft turbulence data described below, the frequency of turbulence reports above the “moderate or greater” threshold is approximately 0.02 %. Likewise, violent tornadoes, wind, and hail events are infrequent. Algorithms that learn with such data must be able to handle the rarity of the class of interest and to properly scale the final predictions to the probabilities represented in nature. We have experimented with both undersampling the majority class or oversampling the minority class (techniques discussed in Weiss and Provost 2003; Johnson et al. 2012) and have found that undersampling the majority class works best. The more balanced data improves the performance of the trees and forest. When outputting actual probabilities, these can be rescaled using methods such as isotonic regression (Zadrozny and Elkan 2002; Niculescu-Mizil and Caruana 2005) or logistic regression.

## Case studies

### Tornadogenesis in Oklahoma

One of the most challenging problems in severe storms forecasting is determining whether or not a supercell thunderstorm will produce a tornado given the characteristics of the storm and surrounding environment. For this study, our aim is to determine the skill of predicting a tornado only with data available from current operational observing systems. Radar-derived supercell tracks in Oklahoma from 1994 to 2003 (Hocker and Basara 2008) were co-located with Oklahoma Mesonet (McPherson et al. 2007) surface observations and gridded reanalysis data from the North American Regional Reanalysis (NARR). The surface observations were used to analyze the storm surface environment and to detect and analyze boundaries while the NARR data provided information about the near-storm environmental conditions above the surface (Fig. 2). The SRRF used these data from the time period of supercell formation until tornadogenesis or storm death to determine the probability of tornadogenesis. More information about the dataset and results with the previous SRRF (without the new enhancements) can be found in Gagne et al. (2012) and McGovern et al. (2011).

Because there are no operational automated probabilistic tornado prediction products on the storm level (the Storm Production Center^4^ has a probabilistic product but it covers an entire day, not a single storm), we have compared the SRRF predictions with meteorological variables that are currently used to assess the tornado potential of a given environment. Table 1 shows the bootstrapped confidence intervals of AUC and binary verification statistics at the threshold that maximizes PSS for each distribution of forecasts. In the table, CAPE stands for Convective Available Potential Energy, which measures the amount of energy in the total atmosphere available to storms. High CAPE is associated with stronger updrafts in storms. BWD is the Bulk Wind Difference, which is the vector difference between winds at the surface and a higher level. Larger BWD means that supercells would have stronger rotation and would be more likely to produce a tornado. SRH (Storm Relative Helicity) is the amount of horizontal rotation available in the lower atmosphere that could be tilted and stretched by supercell updrafts and downdrafts in order to form a tornado. CIN, or Convective Inhibition, is the amount of energy that a parcel of air needs in order to rise. Large magnitudes of CIN prevent storm formation, and small magnitudes of CIN only allow strong isolated storms to form. STP is the Significant Tornado Parameter, an index of tornado potential that is a scaled product of CAPE, BWD, SRH and another value. EHI, the Energy Helicity Index, is the product of CAPE and SRH divided by a constant.

**Table 1:** Comparison of the SRRF against multiple environmental variables used to determine tornado potential. The 95 % bootstrapped confidence intervals (CI) of each verification score are shown. The best score for each parameter is shown in bold

Name	AUC CI	Threshold CI	PSS CI	POD CI	POFD CI	FAR CI
SRRF	**0.65**, **0.66**	0.23, 0.25	**0.21**, **0.23**	0.51, 0.58	0.29, 0.36	0.65, 0.67
CAPE	0.46, 0.52	2463.39, 2985.83	0.08, 0.10	0.22, 0.29	**0.14**, **0.19**	**0.55**, **0.67**
BWD	0.46, 0.52	17.31, 19.58	0.10, 0.13	**0.69**, **0.80**	0.59, 0.67	0.66, 0.74
SRH	0.44, 0.50	139.89, 239.47	0.04, 0.06	0.47, 0.67	0.44, 0.62	0.63, 0.76
CIN	0.53, 0.60	−9.04, −4.63	0.16, 0.19	0.34, 0.44	0.17, 0.26	0.57, 0.65
STP	0.44, 0.50	0.83, 1.78	0.05, 0.07	0.27, 0.39	0.22, 0.33	0.61, 0.72
EHI	0.48, 0.55	1.77, 2.28	0.11, 0.15	0.47, 0.57	0.35, 0.44	0.64, 0.72

The SRRF outperforms the other variables in AUC and PSS, as shown by the non-overlapping confidence intervals. Over the full range of their distributions, all of the parameters except CIN do not have AUC significantly better than random (0.5), but at their optimal thresholds they do show positive skill as indicated by the positive PSS. The SRRF and the other parameters struggle with a high False Alarm Ratio (FAR),^5^ in which one half to three quarters of the tornadic predictions are for nontornadic supercells. The relatively low probability of detection (POD) and high FAR are likely due to the coarse spatial and temporal resolution of the NARR data as well as the fact that neither the SRRF nor the other parameters account for the effects of storm interactions and processes occurring in the mesocyclone. These data are not available operationally.

**Fig. 3:**
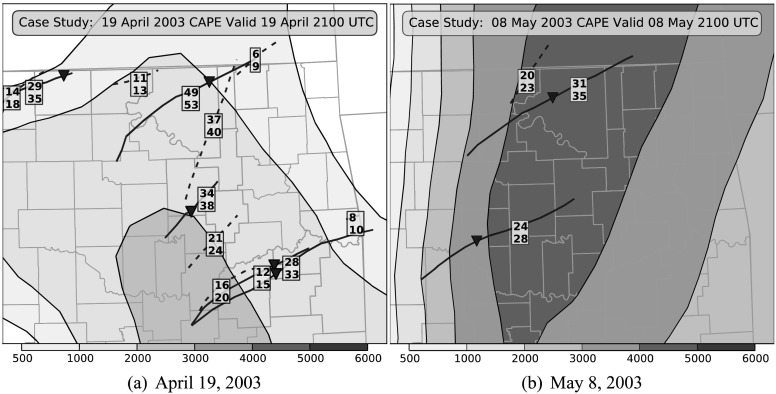
Supercell tracks in Oklahoma on 19 April 2003 and 8 May 2003. *Solid lines* show tornadic supercells tracks, and *dashed lines* show nontornadic supercell tracks. The *triangles* indicate the touchdown site of the strongest tornado associated with each storm. The labels indicate the 95 % confidence intervals (top and bottom numbers) of the SRRF probability of a tornado from each supercell. The *filled contours* show the distribution of Convective Available Potential Energy (CAPE), which is a measure of the environmental instability. It is high in areas with a potential for storms and has sharp gradients along thermodynamic boundaries

The predictions of the SRRF are shown for two separate tornado event days, 19 April and 8 May 2003 (Fig. 3). On 19 April, successive lines of supercells moved through northern and eastern Oklahoma in the afternoon producing multiple brief tornado touchdowns. Because the SRRF is a stochastic algorithm, probabilistic predictions from the SRRFs trained on the same data will vary. The amount of variability is shown by the 95 % bootstrap confidence intervals derived for each supercell. If the user applies the mean optimal decision threshold of 24 % from Table 1, then the SRRF has one miss and one false alarm on April 19 and correctly predicts all of the storms on May 8. On May 8, the SRRF correctly discriminates between the two northernmost storms even though they are in a similar thermodynamic environment. The incorporation of differences in the paths of the storms likely led to the differences in the probabilities. For the clusters of supercells on April 19 (where the tracks are near each other), the SRRF gave the highest probabilities to the southernmost supercell in both instances. That is consistent with the conceptual model of supercell interaction even though the SRRF did not have explicit information about the presence of other supercells in the vicinity. With situations like these, the SRRF enhanced classification ability can provide greater insights for forecasters than the currently used environmental parameters.

Variable importance rankings show significant contributions from attributes of all objects and relations included in the dataset (Table 2). The angle and distance between boundaries and storms were the most important attributes by far. Storm movement at an angle roughly 45^∘^ relative to the boundary can increase the likelihood that individual supercells stay isolated and have a consistent moisture source that is not cut off by nearby storms. Of the environment attributes, Mean Layer Convective Inhibition (MLCIN) was the most important. High magnitudes of MLCIN impede storm formation, and storms moving into those areas may weaken. Attributes describing the storm surface thermodynamics, moisture, and storm movement were highly ranked. Boundary thermodynamics were also considered important. Although only the top 5 most important attributes were shown for space reasons, many more were considered statistically significant.

**Table 2:** Top five variable importance rankings based on 30 SRRFs. *θ*_*e*_ is the equivalent potential temperature, and MLCIN is the Mean Layer Convective Inhibition

Object/Relation	Item type	Attribute name	Mean score	Std. Dev.
Relation	Nearby	Boundary-Relative Angle	37.48	5.65
Relation	Nearby	Distance	24.85	4.11
Object	Storm Surface	*θ*_*e*_	4.53	1.51
Object	Environment	MLCIN	4.38	1.77
Object	Boundary	Pressure	2.85	2.14

### Convectively induced turbulence

One of the hazards to aviation produced by severe weather is convectively-induced turbulence. Unlike convective turbulence, which is produced within the storm itself, convectively-induced turbulence originates in the storm and propagates throughout the surrounding clear air. Because it is neither visible nor measurable on radar, algorithms such as the SRRF are needed to infer, or diagnose it. For this application, the SRRF was trained on measurements of the Eddy Dissipation Rate (EDR) from select United Airlines aircraft from March 18 through June 10 of 2010, a time period when CIT would be expected to be responsible for a significant proportion of turbulence encounters. An EDR threshold of 0.3 m^2^ s^−3^ was used to distinguish Moderate or Greater (MoG) turbulence. The flight data were paired with co-located radar and Weather Research and Forecasting (WRF, Skamarock and Klemp 2008) model data. To balance the training set due to the large number of non-turbulent cases and to keep the training set size computationally feasible, a random sample of up to 15 turbulent and 15 non-turbulent cases were taken from each day for a total of 1365 training cases. One set of SRRFs was trained with only WRF data, while a second set of SRRFs was trained with both WRF data and objects derived from composite radar reflectivity and vertically integrated liquid (VIL) (see Fig. 2). Both versions of the SRRF were compared with Graphical Turbulence Guidance (GTG) predictions derived from the same WRF model. Verification was done on both the deterministic GTG and a logistic-regression-calibrated probabilistic GTG.

The verification statistics for each algorithm, computed on independent testing subsets of the resampled data, are shown in Table 3. Both SRRF models outperform GTG in AUC, PSS, and BSS. The greater skill of the SRRF is likely due to better handling of the convective induced turbulence, which GTG does not handle well. Using just WRF data, including derived turbulence diagnostics developed for GTG, provides very skilled predictions from the SRRF, and the addition of radar data does not change the AUC. At the optimal prediction threshold around 50 %, the WRF and Radar SRRF does provide a more skilled prediction as measured by PSS due to an increase in POD and slight decrease in FAR compared to the WRF SRRF. Since BSS is the mean squared error between probabilistic forecasts and binary observations, it can be used as a proxy for sharpness, or forecast spread. Sharper turbulence diagnoses and forecasts help pilots identify safe routes and are therefore more desirable. Within the distribution of aircraft observations, both SRRFs have similar sharpness and have a greater amount than the GTG.

**Table 3:** Comparison of the bootstrapped 95 % confidence intervals (CI) for multiple verification statistics applied to SRRFs trained on the turbulence cases with just collocated WRF model data and with both WRF model data and nearby radar-derived objects, as well as Graphical Turbulence Guidance (GTG) predictions. The Threshold (Thresh.) refers to the probability or EDR threshold with the highest Peirce Skill Score (PSS). Sharpness refers to the standard deviation of the forecast distribution

Model (Data source)	AUC CI	Thresh. CI	PSS CI	POD CI	FAR CI	BSS CI
SRRF (WRF)	**0.91**, **0.92**	0.49, 0.54	0.68, 0.70	0.79, 0.81	0.11, 0.13	0.54, 0.55
SRRF (WRF, Radar)	**0.91**, **0.92**	0.50, 0.56	**0.70**, **0.72**	**0.81**, **0.84**	**0.10**, **0.13**	**0.54**, **0.56**
GTG Logistic (WRF)	0.85, 0.87	0.47, 0.52	0.58, 0.61	0.76, 0.79	0.18, 0.20	0.39, 0.42
GTG (WRF)	0.85, 0.87	0.23, 0.25	0.58, 0.60	0.75, 0.80	0.18, 0.21	NA

The variable importance scores for the SRRFs trained on WRF and Radar data (Table 4) provide some insight into small difference in AUCs. Most of the top variables are turbulence parameters from the WRF data. The ones chosen all describe different ingredients of turbulence in the environment and have been used in other turbulence models (Kaplan et al. 2006; Sharman et al. 2006). Range and azimuth (relative angle) to nearby rain, convection, hail, and VIL objects, inform the SRRF of proximity to storms that may be generating convective turbulence as well as what temporal changes are occurring over the last 30 minutes. The ability to interpret and derive predictive ability from these relationship attributes gives the WRF and Radar SRRF part of its slight advantage over the WRF SRRF. This additional information also has an effect on the spatial characteristics of the SRRF predictions. This can be verified by looking at a case study.

**Table 4:** Top six important variables based on multiple SRRFs. The NCSU2 Turbulence Index is the cross product of the Montgomery stream function and relative vorticity (Kaplan et al. 2006)

Objection/Relation	Item type	Attribute name	Mean score	SD
Object	Aircraft	Frontogenesis Function	186.9	21.76
Object	Aircraft	EDR	123.8	26.15
Relation	Nearby	Range	92.37	12.70
Object	Aircraft	NCSU2 Turbulence Index	89.41	20.16
Object	Aircraft	Deformation	89.34	21.18
Relation	Nearby	Azimuth	79.39	5.97

**Fig. 4:**
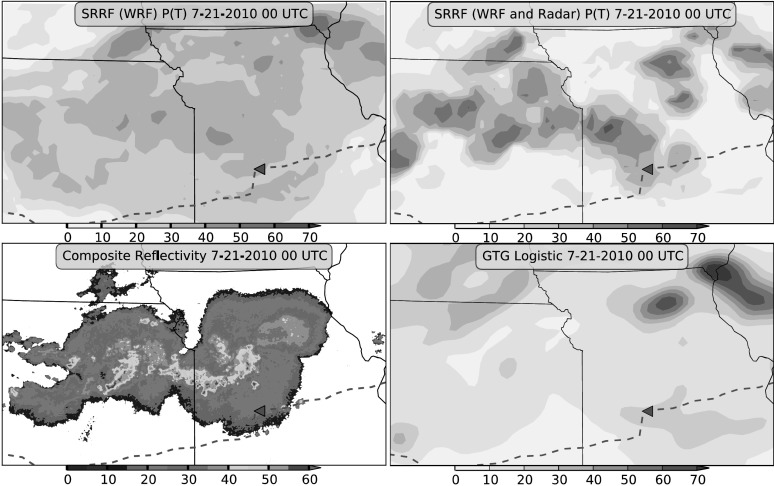
Clockwise from upper left: Probability of moderate or greater (MoG) turbulence from a SRRF trained with WRF data, probability of MoG turbulence from a SRRF trained with WRF and radar data, probability of MoG turbulence from the logistic regression calibrated GTG derived from the WRF, and composite radar reflectivity. The flight path of UAL Flight 967 is indicated by the *red dashed line*, and the location of the turbulence incident is marked with a *triangle*

For the case study evaluation, we produced maps of the SRRF predictions for Kansas and Missouri on 21 July 2010 at 0000 UTC. At 0014 UTC, United Airlines (UAL) Flight 967 experienced severe turbulence in Missouri resulting in multiple injuries. Figure 4 shows the SRRF nowcast of convectively induced turbulence for this case based on co-located WRF data, the SRRF nowcast using the WRF and radar data, logistic regression probabilistic GTG derived from the WRF, and the composite radar reflectivity. At the time of the incident, the plane was located in an area where the WRF SRRF predicted a 30 % chance of moderate or greater turbulence, the WRF and Radar SRRF predicted a 35 % chance, and the GTG predicted at 21 % chance. In this case, the WRF and Radar SRRF produces spatially sharper probabilistic forecasts with higher probabilities around the storms and very low probabilities further away, a highly desirable characteristic for pilots. The WRF SRRF issues a broader area of moderate probabilities. Although the verification scores are similar between the SRRFs, the spatial characteristics differ noticeably, and the influence of the radar data is apparent in the higher probabilities around the radar echoes. High probabilities also appear in areas distant from the strongest radar echoes for all three models where turbulence ingredients analyzed by the WRF have a more dominant role. The GTG logistic prediction is smooth over the storm region and indicates little turbulence near the plane. By filtering the information from WRF and radar data, the SRRF produces a probabilistic prediction that highlights threats from all resolvable turbulence factors. While a longer training and testing set is desirable and calibration of the predictions remains to be done, these promising results suggest that the SRRF could be a valuable component of an operational turbulence diagnosis capability.

## Impact on meteorology

While the occurrence of tornadic supercells remains a relatively rare event, due to their significant destructive qualities and potential for loss of life, they remain at the forefront of research to determine the parameters likely to result in tornadogenesis. Numerous studies have been conducted to identify those environmental conditions that distinguish between storms that produce tornadoes, those that do not, and other severe weather phenomena. The overarching goal has been to increase the confidence of forecasts and warnings while providing increased lead-time to the public to protect lives and property.

Several different approaches have been employed to accomplish this challenging task. One involves recognizing large-scale weather patterns at various heights in the atmosphere that typically are associated with severe weather outbreaks (e.g., Miller 1967). Another approach is to select various environmental parameters, such as storm relative helicity that indicates the likelihood that rotation will develop about a vertical axis or convective available potential energy (CAPE) that is a measure of atmospheric instability that favors the formation of severe thunderstorms (e.g., Johns and Doswell 1992; Moller et al. 1994; Thompson et al. 2007).

Such methods have led to improved forecasting of conditions favorable for supercells and tornadoes. However, tornadoes remain a destructive phenomena that can cause considerable loss of life. For example, in 2011, 553 fatalities occurred in the United States as a result of killer tornadoes. In addition, fatalities occurred within every categorical classification of tornado intensity (i.e., the Enhanced Fujita Scale which ranges from EF0 to EF5).

Because tornadogenesis is a complex process driven by multiple environmental variables, new methods are required to determine the likelihood of tornado formation using limited environmental observations. Similarly, aircraft turbulence is difficult to detect and predict with univariate analyses. However, by using the novel Spatiotemporal Relational Probability Trees and Spatiotemporal Relational Random Forests, one is able to explore the more complicated interrelationships that occur in nature. For example, the two schemas in Fig. 2 show the types of intricate relationships that can exist when one attempts to identify the development of turbulence that will adversely affect aircraft or the factors associated with the formation of tornadoes. By ranking the importance of the variables (see Tables 2 and 4), one is able to determine the combination of variables that will have the most impact on the development of a particular weather phenomenon. In future work, we will also vary the object definitions by perturbing the contour thresholds. This will help to remove any preconceived human bias.

## Fielding the techniques

A new concept has been proposed to further increase the lead time for issuing tornado and severe storm warnings. This concept, called Warn-on-Forecast (WoF, e.g., Stensrud et al. 2009), assimilates radar and other observational data into a high-resolution numerical model. It is proposed that by running the numerical model forward in time, advanced warnings can be issued based on the time and location of simulated tornadic storm development. This warning approach is expected to become operational within 10 years. The anticipated role of SRRF in this effort will be to identify the evolving relationships within the numerical storms that provide an early indication that a tornado will be developing. These relationships then can be incorporated into the WoF model to help provide an earlier indication that within-storm conditions are becoming favorable for tornado formation. Every spring, weather forecasters from across the nation are brought to the Hazardous Weather Testbed in Norman, OK to help evaluate the usefulness of cutting-edge forecasting techniques and to provide input on improvements (e.g., Clark et al. 2012). Once the WoF approach with contributions from the SRRF is completely developed, it will be evaluated and refined in the testbed for a year or two before being deployed to weather forecast offices.

The FAA Aviation Weather Research Program funds the National Center for Atmospheric Research (NCAR) to develop improved turbulence forecasting and nowcasting technologies to support aviation users. Each new version of the Graphical Turbulence Guidance (GTG) system is independently evaluated to verify its improved capabilities and accuracy (e.g., Wandishin et al. 2011); when approved by the FAA, it is operationally deployed at the NWS Aviation Weather Center for incorporation into the Aviation Digital Data Service (ADDS; aviationweather.gov/adds). An initial GTG Nowcast (GTG–N) product is currently under development at NCAR to provide 15-minute-update “snapshots” of turbulence within GTG, including a CIT diagnostic capability as described in Williams (2013). The SRRF method could provide an enhancement of this CIT diagnosis capability, either as a replacement for the existing random forest approach or as an additional input module. This would require transitioning the SRRF software to NCAR, performing training and evaluation on larger, more recent datasets, and evaluating cost versus benefit for the overall GTG-N system, including runtime, resource use and system complexity. In the future, GTG-N may incorporate on-line training for some predictive modules, and the SRRF would be an ideal candidate for performing that function for CIT.

The use of SRRFs for the investigation of convectively-induced turbulence has several benefits over the random forest (RF) approach described in Williams et al. (2008), Williams (2013). For example, the RF approach relies on computing local statistics of various predictor fields around the aircraft location at various radii, since the scales of influence are not known a priori. It utilizes horizontal and vertical distances to various contours, but is not able to jointly utilize information about the properties of the objects defined by those quantities. In contrast, the SRRF requires a definition of objects, attributes and relations via a schema, but is more flexible in exploring the possibly predictive relationships without requiring pre-defined thresholds or statistics. Moreover, the SRRF’s object-oriented approach lends itself to knowledge discovery in a form more accessible to scientists, since reasoning from objects, their attributes, and relationships between them is more consistent with humans’ heuristic models of physical processes.

The end result of examining the complex relationships in this way is greater understanding of the physical variables and their relative importance. From a scientific standpoint, this promotes new research that can be tested through improved observation collection strategies, case studies, and theoretical constructs. Further, humans use such information as part of their subjective approach to forecasting turbulence and severe weather via improved overall understanding of the key physical constraints that drive the development of turbulence and tornadogenesis.

## Lessons learned in interdisciplinary collaboration

Embedding machine learning and data mining techniques in the domain science field of meteorology has provided us with a variety of lessons, both of a general nature and some specific to machine learning. We describe each of these below, hoping that they will help other machine learning researchers who want to work closely in interdisciplinary collaboration. In order to really work on “machine learning that matters” (Wagstaff 2012), we are not working on surface collaborations but true interdisciplinary research.

When scientists of different disciplines begin to work together, they must first learn to speak each other’s language. This does not refer to the verbal language such as English but rather to the language of science used to communicate ideas to one another. At the surface, it seems as though scientists should be able to communicate easily. But when we begin describing terms to one another, we discover that sometimes words mean two different things in two different disciplines. For example, the word *object* has a very specific meaning in relational learning. It also has a meaning in meteorology, and they are not the same.

In addition to words that may mean different things, the scientists in both disciplines need to understand the technical terms of the other discipline. For example, this paper defined a number of technical meteorological terms such as vorticity and gravity waves. We assume that the computer science reader did not necessarily know these terms. In order to work closely with the meteorologists, the computer scientists had to learn these items. Likewise, the meteorologists have learned many technical terms for machine learning and data mining.

Once the language issue is resolved, the next lesson learned is to identify the real scientific question that the domain scientists are trying to answer. Often when the problem is initially described, the very specific scientific question that is being asked is not clear. This requires clear communication back and forth. It is critical because otherwise the method developed will not be of use.

Machine learning researchers often evaluated techniques using measures such as AUC or even accuracy, depending on the domain. While meteorologists use general statistics to evaluate their techniques, fielding a new technique requires a focus on case studies. It is critical that both sides of an interdisciplinary research team know what it means for the technique to be successful. This enables the technique to actually be adopted and also enables both sides to publish the results.

Another difference between machine learning and a domain science such as meteorology comes in the form of the solution desired. In many cases, the goal of a machine learning method is to have the best evaluation score. In some ML applications to severe weather, the black box technique with a high score will be the best answer. For turbulence, this is a possibility for improving automated generation of forecast grids. However, for many applications, a human forecaster needs to deeply understand the technique or the model before it will be used. Tornado warnings are issued by humans, not by a computer. If the forecaster does not understand the model, they are very unlikely to use it. The key lesson here is to know what form of a solution is needed to adopt the technique.

Another lesson that we have learned is to not allow the assumptions of either side of the research to constrain the solution. Sometimes one domain is convinced that the problem is not solvable and then constrains the question being investigated, which constrains the solution. This can happen either from the computer scientists or the domain scientists. It is important to not let current solutions or techniques constrain the question being asked and thus constrain the solution.

## Ongoing and future work

We have been working on severe weather prediction using spatiotemporal machine learning and data mining for over eight years and this paper summarizes our most recent approach. Given the nature of this special issue on machine learning for science and society, we specifically focused on two case studies in this paper: one for predicting tornadoes in Oklahoma and one on predicting aircraft turbulence. In current work, we are developing a novel set of high-resolution simulations of supercell thunderstorms that are capable of resolving tornadoes and turbulence. These simulations are at a 75 m horizontal resolution and the domain is 125 km by 125 km by 20 km. These simulations are unique as no one else has generated such a data set of simulations at this fine-scale resolution. When we complete the simulations, they will provide a distinctive data set to examine tornadogenesis and convectively-induced turbulence. Figure 1(a) shows the near-surface reflectivity from one of our simulations. The SRRF methodology will be an important tool for knowledge discovery in analyzing this dataset.

The SRRF turbulence predictions may also be implemented as a real-time component of a turbulence nowcast system currently being developed at NCAR under sponsorship of the FAA. The system, know as the Graphical Turbulence Guidance Nowcast (GTGN), utilizes a component for diagnosis of turbulence in and around thunderstorms. An additional possibility would be to include automated training to update the SRRF using recent data. This could help the system deal gracefully with changing inputs, e.g., changes to the operational numerical weather prediction model, satellite, or radar products, or with changes in synoptic weather patterns such as those associated with the El Niño-Southern Oscillation.

The enhanced SRRF was able to improve prediction over the older versions by making use of the new ability to distinguish arbitrary shapes using shapelets. We are also enhancing it with the ability to autonomously discover relationships, as described above. However, this discovery currently only works with 3D data and the data presented here was 2D. We are developing 3D approach and we expect the 3D approach to be valuable in our tornado and turbulence simulations, which provide full 3D pictures of the atmosphere every 30 seconds. We are also developing approaches to improve the prediction of severe wind and hail events.

**Reproducibility of research**: In conjunction with the publication of this paper, we have released the full SRPT/SRRF code and the turbulence and tornadogenesis data in the format used by our algorithm at http://idea.cs.ou.edu/software/.

## Figures and Tables

**Algorithm 1:**
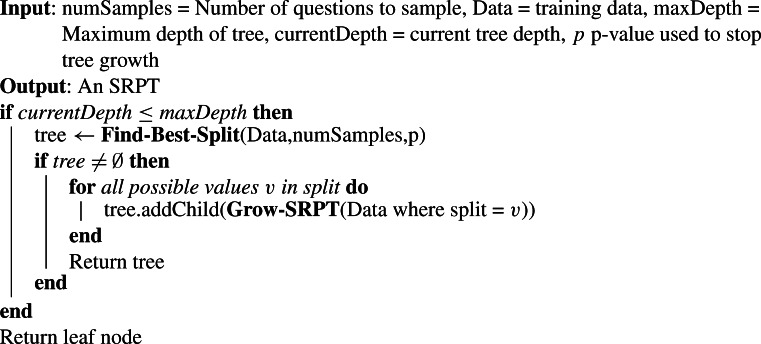
Grow-SRPT

**Algorithm 2:**
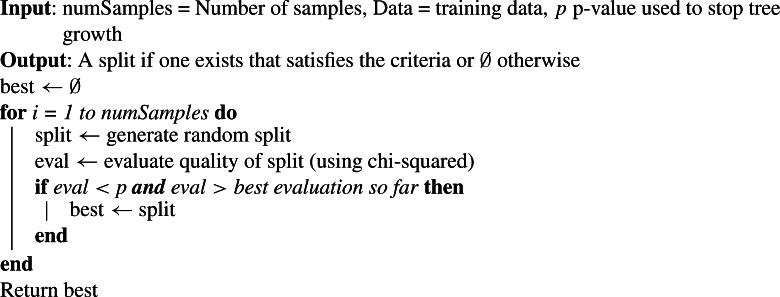
Find-Best-Split

## Appendix A: Details of Spatiotemporal Relational Probability Trees

Spatiotemporal Relational Probability Trees (SRPT) are trained using the standard greedy algorithm for decision trees, e.g., the one used for ID3 (Quinlan 1986). Since the trees are used in a forest, they are not pruned. Pruning could be implemented if a single tree would be used instead. The algorithm for growing the SRPTs is given in Algorithm 1.

The algorithm proceeds by greedily finding the best split possible at each level of the tree. The primary difference between the traditional decision tree growing algorithm and the one used to grow the SRPT is that there is essentially an infinite number of possible questions that can be asked at each tree split. We handle this by sampling from the set of possible questions. The best question is chosen from a set that is sampled. The possible questions are given as a template and we sample from the training data to fill in the variables in the template. This shows up in Algorithm 2 when we generate the random split.

The full set of possible questions are given below. Variables to be filled in using sampling are denoted using italics. The questions are grouped by type. If a question refers only to an object or relationship, it is noted as such. If the question can refer to either an object or relationship, we denote it as an item. If the question has a choice, such as the statistic to be chosen, that is also done when it is created. The choice of a statistic is uniformly random from the choices listed in all capitals.

Although the questions look as if they are binary, simple yes/no questions, we actually split the data in three ways. Because a single graph is not required to have all of the types of objects or attributes, and an object is not even required to have all the attributes that it could possibly have as specified by the schema, we have three branches. The first is the yes branch, meaning the graph matched the question. The second is the no branch, meaning the graph had the attribute and or item mentioned but it did not match. The third is the error branch, meaning the graph did not have the item or attribute mentioned.

The majority of the questions are spatiotemporal. The following three question templates are the non-spatiotemporal questions.

- Does an item of type *t* exist in the graph?
- Does an item of type *t* have a scalar attribute *a* with value ≥ *x*?
- Does an item of type *t* have a scalar attribute *a* with value = *x*?

The following questions split the data on temporal characteristics on temporal data. Some of the questions deal with the temporal data directly and some of them look at statistics on the data.

- Does an item of type *t* exist in the graph for at least *x* steps?
- Does an item of type *t* have ANY or ALL value in a 2D temporal attribute *a* with value ≥ *x*?
- Does an item of type *t* have ANY or ALL value in a 2D temporal attribute *a* with value = *x*?
- Does an item of type *t* have a value in a 2D temporal attribute *a* with value ≥ *x* for at least *s* time steps?
- Does an item of type *t* have a value in a 2D temporal attribute *a* with value ≤ *x* for at least *s* time steps?
- Is the partial derivative (computed using finite differences) of a 2D temporal attribute *a* on item of type *t* ≥ *x* within *s* time steps?
- Does an item of type *t* have a 2D temporal attribute *a* where the MEAN or MAXIMUM or MINIMUM standard deviation is ≥ *x*?

The following questions split the data on either spatial or spatiotemporal characteristics. These work on fielded attributes. Fields can have either scale values or vectors. Fields can be 2-D or 3-D. Vectors can also be 2-D or 3-D. If a particular question requires a specific structure, such as 3-D only, that is noted.

Because we work with weather data, we added several questions that specifically deal with wind vectors. Wind vectors can either be 2-D or 3-D. The full wind vector has three components 〈*u*,*v*,*w*〉. The first component is the wind in the East-West direction, the second component is the North-South direction, and the third component represents the vertical wind. The formulas below refer to 〈*u*,*v*,*w*〉 for wind and 〈*x*,*y*,*z*〉 for the underlying grid. X and Y are orthogonal horizontal directions and Z is vertical. Derivatives are calculated using finite differences.

- Does an item of type *t* with scalar field attribute *f* have ANY/ALL value ≥*x*?
- Does an item of type *t* with scalar field attribute *f* have ANY/ALL value = *x*?
- Is there an item of type *t* with a scalar field attribute *f* where the maximum magnitude of the gradient is ever ≥ *x*?
  - For 3-D scalar fields: $\langle\frac{df}{dx}, \frac{df}{dy}, \frac{df}{dz} \rangle$
  - For 2-D scalar field: $\langle\frac{df}{dx}, \frac{df}{dy} \rangle$
- Is the MEAN/MAXIMUM/MINIMUM/STANDARD DEVIATION of scalar field attribute *f* on item of type *t* ≥ *x* at ANY/ALL time steps?
- Is there an item of type *t* with a 2-D or 3-D wind vector field attribute *a* with the maximum *divergence* ≥ x or minimum *convergence* ≤ *x* (at a z-level z)?
  - Divergence is $\frac{du}{dx} + \frac{dv}{dy}$ and convergence is the negative of divergence (Glickman 2000). *u* is the x-component of the 3D wind vector and *v* is the y-component.
- Is there an item of type *t* with a 2-D or 3-D wind vector field attribute *a* with the MAXIMUM/MINIMUM of the shearing/stretching deformation ≥ *x* (at a z-level z)?
  - Shearing/stretching deformation of a flow field is a change in the direction and speed of a flow owing to shearing (where speed changes perpendicular to the flow direction) and/or stretching (where speed changes along the flow direction) in the flow.
  - Shearing deformation is $\frac{dv}{dx} + \frac{du}{dy}$
  - Stretching deformation is $\frac{du}{dx} - \frac{dv}{dy}$.
- Is there an item with a 3D wind vector field attribute *a* with the MAXIMUM/MINIMUM magnitude of the average horizontal vorticity ≥/≤ *x*?
  - Vorticity is a vector.
    $$\biggl\langle\frac{dw}{dy} - \frac{dv}{dz}, \frac{du}{dz} - \frac{dw}{dx}, \frac{dv}{dx} - \frac{du}{dy}\biggr\rangle. $$
    Horizontal vorticity is the first two components of this vector and vertical vorticity is the third component and *w* is the z-component of the 3D wind vector.
- Is there an item with a 3D wind vector field attribute *a* with an average horizontal vorticity vector direction within (+/−) 22.5 degrees of the 16 compass points (N/NNW/NW/WNW/W/etc)?

The following questions all examine the shape of the data. Shapes can be extracted from 2-D or 3-D fields. Following on Keogh et al.’s work (Ye and Keogh 2009; Mueen et al. 2011), we can also identify shapes in temporal data using shapelets. We use their description of how to compute them efficiently to precompute the statistics as the data is being loaded in. This enables the shapelet questions to be evaluated very quickly. In our implementation, the pre-computation does not add noticeable overhead.

In order to detect shapes in 2-D fields, we reduce the fields to a single temporal array following Keogh’s method. This is done by identifying the centroid of the 2-D field and detecting the outline. The distance from the centroid to the outline all the way around the shape becomes the temporal array.

- Does the shapelet *s* of temporal array attribute *a* on item of type *t* match in this graph?
- Given a shape distribution *d* (described in Sect. 3.2), is the shape distribution of the 3-D fielded attribute *f* on item of type *t* statistically the same?
  - Following Osada’s paper we use D2 (distance between 2 randomly chosen points) as the shape distribution and the L1 norm to compute the distance between the pdfs.
- Given two different shape distributions *d1* and *d2*, does the shape distribution of the 3-D fielded attribute *f* on item of type *t* change from *d1* to *d2* within time period *t*?
- Given a shape distribution *d*, does the shape distribution of the 3-D fielded attribute *f* on item of type *t* stay statistically the same as *d* for at least *t* steps?

Conjunctive questions can combine the results of the base question in interesting, and possibly spatiotemporal, ways. All of the questions detailed above are base questions. The following questions are all conjunctive questions.

- Are there at least *n* matching items to base question *q*?
- Are items matching base question *q1* in a temporal relationship (listed below) with items matching base question *q2*?
  - Allen (1991) introduced a full set of temporal relations that any two events can have. Because they are symmetric and we are sampling from the data, we only take half of the relations. The other half can be found in a different sample. The possible relations are: before, meets, overlaps, equals, starts, finishes, and during
- Are there objects matching fielded question *q1* that are within Euclidean distance *d* of objects matching fielded question *q2* for at least *t* steps?
  - This can only apply to objects that have a fielded attribute. The location of the center of the field is used to compute distance.
- Is there an object matching fielded question *q1* that is in a spatial relationship with an object matching fielded question *q2* for at least *t* steps?
  - The spatial relationship is measured using the method described in Sect. 3.2, which is derived from the shape distributions.

Before the tree building begins, we must process the data. Since we often run many forest building algorithms in parallel with different parameters, we preprocess the data the first time it is read in. When we preprocess the data, we create a number of new attributes that were not specified in the original schema. These are added to the schema and used to compute statistics and to enable interesting questions to be evaluated efficiently. These dynamically created attributes are listed below.

- For each scalar field, we create two new temporal array attributes representing the maximum and minimum magnitude of the gradient for that attribute over time.
- For each wind vector field, we create two new temporal array attributes representing the maximum and minimum of the divergence/convergence over time. Note, this is currently only computed for z-level 0 but it can easily be extended.
- For each wind vector field, we create two new temporal array attributes representing the maximum and minimum of the deformation over time. Note, this is currently only computed for z-level 0 but it can easily be extended.
- For each 3-D wind vector field, we create two new temporal array attributes representing the maximum and minimum of the horizontal vorticity over time. Note, this is currently only computed for z-level 0 but it can easily be extended.
- For each object that has a 2-D field associated with it, create an associated temporal array attribute representing the shape using Keogh’s method as described above.
- For each object with a scalar field, create a temporal array for each of the MEAN/ MAXIMUM/ MINIMUM/ STANDARD DEVIATION of the values in the field at each time step.
- For each object with the 2-D field, create a temporal attribute of the area of that field.
- For each object with a 3-D field, create a temporal attribute of the average of the area across all z-levels.
- For each object with a 3-D field, create a temporal attribute of the volume at each time step.

## Appendix B: Verification statistics

The Relative Operating Characteristic (ROC) curve (Mason 1982) is created by evaluating a set of forecasts over a range of thresholds. A contingency table is created at each threshold. Multiple summary statistics can be derived from the contingency table shown in Table 5. Box *a* represents the hits, box *b* represents the false alarms, box *c* represents the misses, and box *d* represents the true negatives *n*. The statistics used in the paper are shown in Table 6. Probability of Detection (POD), also known as the hit rate or true positive rate, is the proportion of observed yes events that are correctly forecast. The Probability of False Detection (POFD), also known as the false positive rate, is the proportion of no events that were incorrectly forecast. The False Alarm Ratio (FAR), is the proportion of yes forecasts that were observed to be no events. The Peirce Skill Score (PSS; Peirce 1884) is the difference between the POD and the POFD.

**Table 5:** Example binary contingency table

		Observed	
		Y	N
Forecast	Y	a	b
	N	c	d

**Table 6:** Scores derived from the binary contingency table

Name	Abbreviation	Formula
Probability of Detection	POD	$\frac{a}{a+c}$
Probability of False Detection	POFD	$\frac{b}{b+d}$
False Alarm Ratio	FAR	$\frac{b}{a+b}$
Peirce Skill Score	PSS	POD-POFD

The Brier Score (Brier 1950) has been used to verify the both the calibration and refinement of probabilistic forecasts (Murphy 1973). In its simplest form (Eq. (1)), it is the mean squared error of probabilistic forecasts versus binary observations. The Brier Skill Score compares the BS of a forecast with the BS of the climatological probability (Eq. (2)). The calibration aspect of BS and BSS can be used as a proxy to measure the sharpness of a forecast.

1

2

## Footnotes

1 Vorticity is a measure of the local rotation about an arbitrarily-oriented axis.

2 The tropopause is a layer 10 to 15 km above the ground that separates the troposphere below (where temperature decreases with height) from the stratosphere above (where temperature increases with height).

3 Gravity waves are undulating waves of air in the atmosphere that alternately overshoot and undershoot an equilibrium level. The waves come from an interaction between gravity and the buoyancy of air.

4
http://www.spc.noaa.gov.

5 All of the verification statistics are defined in Appendix B.
